# Supplementary material for: Adaptive diagnostic reasoning framework for pathology with multimodal large language models
Source: Commun Med (Lond). 2026 Mar 7;6:236. doi: 10.1038/s43856-026-01491-z (PMC13096528; doi:10.1038/s43856-026-01491-z)
Supplement: Supplementary file 2 — SI Figures [file 43856_2026_1491_MOESM2_ESM.pdf]

Initial generation prompt

```
# Task
What attributes does this pathology image show? Try to generate highly discriminative attributes that can help
→ identify the type of the tumor.
```

Classification prompt

```
# Task
Determine what kind of breast tumor this image shows from the following options based on the context and other
→ information... Answer the letter A or B as prediction...

Text: The image shows the following features: ...
The answer is:
```

Error collection prompt

```
## Image 1
Text: The image shows the following features: ...
Label: Normal
Prediction: Invasive Carcinoma.

## Image 2
Text: The image shows the following features: ...
Label: Invasive Carcinoma
Prediction: Normal.

...
```

Fail reason hypothesizing prompt

```
I'm trying to write a prompt used to generate description for the given image, where the generated description
→ can enhance the image zero-shot classification of breast tumor in the image.
The two types of breast tumors are: A. Normal, B. Invasive Carcinoma.

My current prompt is: ...

But this prompt gets the following examples wrong: ...

Give a reasons why the prompt could have gotten these examples wrong.
```

New prompts hypothesizing prompt

```
I'm trying to write a prompt used to generate description for the given image, where the generated description
→ can enhance the image zero-shot classification of breast tumor in the image.
The two types of breast tumors are: A. Normal, B. Invasive Carcinoma.

My current prompt is: ...

But this prompt gets the following examples wrong: ...

Based on these examples the problem with this prompt is that ...

Based on the above information, I wrote {steps_per_gradient} different improved prompts.
Each prompt is wrapped with <START> and <END>.

The {steps_per_gradient} new prompts are:
```

Fig. S1. Prompt examples for autonomous diagnostic learning. Example prompts used in a single round of image description strategy refinement. The first prompt requests a descriptive account of a given image. The second prompt leverages this description to generate a diagnostic classification. Following classification, error cases are collected, with the LLM articulating potential reasons for misclassification. Finally, the last prompt incorporates this feedback to propose revised image description strategies and diagnostic guidelines.

(A)

Prompt used for N/IC classification:

You are assisting with a breast cancer pathology image classification task. The goal is to distinguish normal breast tissue from invasive breast carcinoma in H&E-stained microscopy images. Below is the clinical and morphological context you must rely on.

Normal Breast Tissue:
- Normal breast tissue is composed of terminal duct–lobular units (TDLUs), which contain small acini arranged into lobules.
- Ducts and acini have a dual cell layer: an inner luminal epithelial layer and an outer myoepithelial layer. The myoepithelial layer is a key indicator of non-invasive epithelium.
- Surrounding stroma contains a mixture of fibrous connective tissue and adipose tissue.
- Morphological features include well-organized architecture, intact myoepithelial layers, uniform small nuclei with low atypia, low mitotic activity, and the absence of desmoplastic stromal reaction.

Invasive Breast Carcinoma:
- Invasive breast carcinoma is defined clinically as malignant epithelial cells that have breached the basement membrane and infiltrated the surrounding stroma.
- This distinction directly affects cancer staging, prognosis, and treatment decisions.
- Key morphological features of invasive carcinoma include: loss of the normal duct/lobular architecture; absence of the myoepithelial cell layer around tumor structures; infiltrative patterns such as irregular glands, nests, cords, or single tumor cells invading stroma; marked cytologic atypia such as enlarged pleomorphic nuclei and prominent nucleoli; increased mitotic figures; and the presence of desmoplastic (fibrotic) reactive stroma.
- Common invasive patterns include malformed ducts with irregular lumens, solid nests or sheets of tumor cells, trabecular arrangements, or single-file infiltration.

Task Relevance:
- Determining whether a region is normal or invasive is the most fundamental and clinically meaningful decision in breast pathology.
- For machine learning models, highly discriminative cues include cell density and pleomorphism, architectural irregularity, loss of the myoepithelial layer, desmoplastic stroma, and abnormal gland formation.

(B)

Prompt used for DCIS/IC classification:

You are assisting with a breast pathology image classification task. The goal is to distinguish ductal carcinoma in situ (DCIS) from invasive carcinoma (IC) in H&E-stained microscopy images. Below is the clinical and morphological context you must rely on.

Ductal Carcinoma In Situ (DCIS):
- DCIS consists of malignant epithelial cells that remain confined within the mammary ducts.
- The basement membrane is intact, and a myoepithelial cell layer surrounds the ducts. This intact myoepithelial layer is a key diagnostic hallmark.
- Tumor cells fill or partially fill ducts without extending into the surrounding stroma.
- Ducts may be dilated or distended.
- Architectural patterns include comedo, cribriform, solid, papillary, and micropapillary.
- Cytologic atypia is present but restricted to intraductal spaces.
- Comedo necrosis may be seen.
- No desmoplastic stromal reaction is present because invasion has not occurred.

Invasive Carcinoma (IC):
- IC is defined by malignant epithelial cells breaching the basement membrane and infiltrating the surrounding stroma.
- The myoepithelial layer is absent around invasive tumor structures.
- Tumor growth appears as irregular glands, nests, cords, or single tumor cells invading the fibrous stroma.
- Normal duct architecture is disrupted or destroyed.
- Often accompanied by desmoplastic stromal reaction (fibrotic response).
- Increased cytologic atypia and mitotic activity.
- Common invasive patterns include abnormal tubule formation, solid nests, trabecular arrangements, or single-file infiltration.

Key Distinguishing Criteria:
- DCIS: intact basement membrane, preserved myoepithelial layer, malignant cells confined within ducts, no stromal invasion, no desmoplastic reaction.
- IC: basement membrane breached, myoepithelial layer absent, tumor cells infiltrating stroma, architectural destruction, desmoplastic response present.

Fig. S2. See caption on the next page.

(C)

Prompt used for DCIS/IC classification:

You are assisting with a breast pathology image classification task. The goal is to distinguish among three categories in H&E-stained microscopy images: Normal breast tissue (N), Ductal Carcinoma in Situ (DCIS), and Invasive Carcinoma (IC). Use the following clinical and morphological definitions to ground your reasoning.

Normal Breast Tissue (N):
- Normal tissue consists of terminal duct–lobular units (TDLUs) composed of small acini arranged into lobules.
- Ducts and acini have a dual-layer structure: an inner luminal epithelial cell layer and an outer myoepithelial cell layer.
- The basement membrane is intact and undisturbed.
- Architecture is well-organized, with uniform nuclei, low atypia, low mitotic figures, and no stromal desmoplasia.
- Surrounding stroma contains a mixture of fibrous connective tissue and adipose tissue.

Ductal Carcinoma in Situ (DCIS):
- DCIS is a non-invasive proliferation of malignant epithelial cells confined within the mammary ducts.
- The basement membrane remains intact, and the myoepithelial layer is preserved around ductal structures.
- Tumor cells fill or partially fill the ducts without breaching the basement membrane.
- Ducts may be dilated or expanded.
- Architectural patterns may include comedo, cribriform, solid, papillary, or micropapillary growth.
- Cytologic atypia is present, and comedo necrosis may be observed.
- No invasion into the surrounding stroma and no desmoplastic reaction.

Invasive Carcinoma (IC):
- IC is defined by malignant epithelial cells that have breached the basement membrane and infiltrated into the surrounding stroma.
- The myoepithelial cell layer is absent around invasive tumor structures.
- Growth forms include irregular tubules, nests, cords, sheets, or single tumor cells infiltrating fibrous stroma.
- Native duct/lobular architecture is disrupted or destroyed.
- Often associated with desmoplastic stromal reaction (fibrotic response), increased cytologic atypia, and mitotic activity.

Key Distinguishing Criteria:
- Normal (N): intact architecture with dual epithelial+myoepithelial layers, no atypical proliferation, no stromal invasion.
- DCIS: malignant cells confined within ducts; intact basement membrane and myoepithelial layer; no stromal invasion or desmoplasia.
- IC: basement membrane breached; no myoepithelial layer; tumor cells infiltrating stroma with desmoplastic reaction and loss of normal architecture.

Using this information, when given an image, identify which morphological features are present and determine whether the image belongs to Normal tissue, DCIS, or Invasive Carcinoma.

Fig. S2. Baseline prompts used for in-context learning. Manually curated clinical definitions and morphological features used for the (A) Normal vs. Invasive Carcinoma (N/IC), (B) Ductal Carcinoma In Situ vs. Invasive Carcinoma (DCIS/IC), and (C) Multiclass (N/DCIS/IC) classification tasks. These descriptions establish a static knowledge baseline for comparison with the autonomously optimized RECAP-PATH criteria.

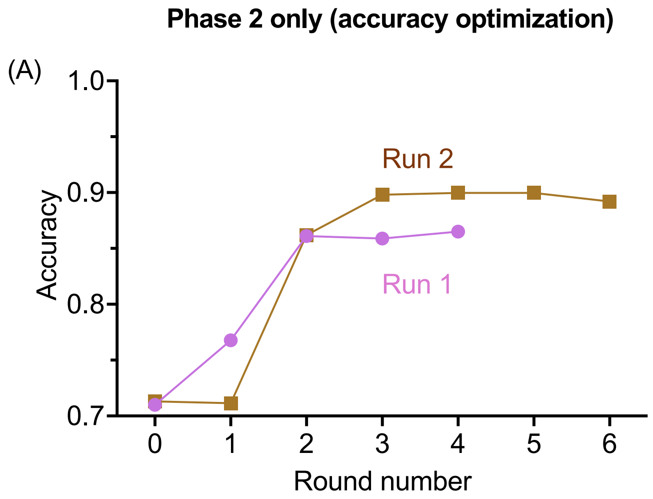

(B)

Example prompt of diagnosis guidelines:
Describe the key histological features of this breast tissue image, including the arrangement of glands, cellular characteristics, and stromal appearance. Highlight features that would indicate either normal breast tissue or invasive carcinoma, paying particular attention to architectural disarray, nuclear atypia, and infiltrative growth patterns. What features support a diagnosis of normal breast tissue?

Example description of Invasive carcinoma:

Certainly! Let's break down the histological features of this breast tissue image.

Key Histological Features:
* Glands: The primary feature observed is a well-defined structure that appears to be a duct or gland…
* Epithelial Cells: The cells lining the gland show a relatively uniform appearance with basally located nuclei. They seem to be cuboidal to columnar in shape.
* Stroma: The surrounding tissue (stroma) appears to be composed of collagenous connective tissue. There are some sparsely distributed ...

Features Suggesting Normal Breast Tissue:
* Organized Glandular Architecture:** The gland maintains a regular shape and arrangement. There's no significant architectural disarray or crowding.
* Uniform Epithelial Cells: The cells lining the gland are relatively uniform in size, shape, and nuclear appearance. There is no prominent nuclear atypia (abnormal variations in nuclear size, shape, or staining).
* Intact Basement Membrane: The epithelial cells are likely supported by an intact basement membrane, which is not directly visible but inferred from the organized structure.
* Non-Infiltrative Growth Pattern: The gland does not appear to be invading the surrounding stroma.

Features That Would Indicate Invasive Carcinoma (and are NOT seen here):
* Architectural Disarray: Irregularly shaped and sized glands, loss of normal ductal patterns, and crowding of cells.
* Nuclear Atypia: Enlarged, hyperchromatic (darkly stained), and pleomorphic (varied in shape) nuclei. Increased mitotic activity (cell division).
* Infiltrative Growth Pattern: Tumor cells invading the surrounding stroma, breaking through the basement membrane, and forming irregular nests or single cells infiltrating the stroma.
* Desmoplasia: A dense, fibrotic reaction in the stroma surrounding the tumor cells.

Diagnosis of Normal Breast Tissue:
Based on the features highlighted, the image is more consistent with normal breast tissue. The organized glandular architecture, uniform epithelial cells, and lack of infiltrative growth patterns support this diagnosis.
Disclaimer: This analysis is based solely on the provided image and should not be considered a definitive diagnosis. A complete diagnosis requires evaluation of the entire tissue sample and correlation with clinical information.

Fig. S3. Results of accuracy-only optimization. (A) Optimizing solely for classification accuracy yielded performance comparable to two-phase learning. (B) However, the resulting diagnostic prompts lacked the depth and structure introduced by the diversification phase, leading to image descriptions that were less detailed and comprehensive than those produced with two-phase optimization.

(A)

Condition	Test 1	Test 2	Test 3	Test 4	Test 5	Test 6	Test 7	Test 8	Test 9	Test 10	Variance
Initial prompt	0.7815	0.7125	0.7500	0.7750	0.7125	0.6750	0.7625	0.7375	0.7500	0.7625	3.22%
Optimized prompt	0.9250	0.9250	0.9000	0.9375	0.9125	0.9625	0.9250	0.9375	0.9250	0.9125	1.63%

(B)

Description 1:
Here are some attributes that can be discerned from the pathology image, focusing on features that might help identify the tumor type:

Cellular Features:
* **Nuclear Morphology:** The nuclei appear relatively uniform in size and shape within the epithelial cells lining the duct. There is no obvious pleomorphism (variation in size and shape) or hyperchromasia (increased staining intensity) that would be strongly indicative of malignancy. The nuclei are basally located.
* **Cytoplasm:** The cytoplasm of the epithelial cells appears well-defined and eosinophilic (pink-staining).
* **Mitotic Activity:** Mitotic figures (cells undergoing division) are not readily apparent in this field of view. The absence of frequent mitoses suggests a lower proliferative rate.

Architectural Features:
* **Glandular/Ductal Structure:** The image shows a well-formed ductal structure with a clear lumen. The epithelial cells are arranged in a single layer along the basement membrane. The duct appears somewhat elongated.
* **Basement Membrane:** The basement membrane appears intact and continuous, which is typical of benign lesions.
* **Cellular Arrangement:** The cells are arranged in a polarized fashion, with the nuclei located towards the base of the cells and the apical surface facing the lumen.
* **Infiltration:** There is no evidence of infiltration into the surrounding stroma.

Stromal Features:
* **Stroma:** The surrounding stromal tissue appears fibrous and contains collagen. There are some cells scattered within the stroma, potentially fibroblasts or inflammatory cells (lymphocytes can be seen).
* **Vascularity:** There are some blood vessels visible in the stroma.

Overall Impression:
Based on these attributes, the image is more suggestive of a benign lesion. The well-defined ductal architecture, uniform nuclei, intact basement membrane, and lack of significant mitotic activity or stromal invasion are characteristic of benign conditions.

Further Considerations (Things that would require more information or different stains):
* **Immunohistochemistry:** Staining for markers like Ki-67 (proliferation marker), ER/PR (estrogen/progesterone receptors), and HER2 would provide additional information about the proliferative rate, hormone receptor status, and potential for targeted therapies.
* **Myoepithelial Layer:** Is there a myoepithelial layer surrounding the duct? Its presence would strongly suggest a benign lesion.
* **Multifocality/Multicentricity:** Is this a single lesion, or are there multiple similar lesions present?
* **Clinical Context:** Patient history, physical exam findings, and imaging results would be necessary for a definitive diagnosis.

Disclaimer: This is a visual interpretation based on a single image. A definitive diagnosis requires a thorough evaluation by a qualified pathologist, considering all available clinical and pathological information.

==

Description 2:
Here are some attributes that can be identified in the pathology image, focusing on features that can help characterize the tissue and potentially identify the type of tumor:

Epithelial Structures:
* **Presence of Ducts/Glands:** The image clearly shows duct-like or glandular structures lined by epithelial cells. This is a key feature, suggesting an origin from glandular tissue (e.g., breast, salivary gland).
* **Epithelial Cell Morphology:**
    * **Cell Shape:** The cells lining the ducts appear to be columnar or cuboidal.
    * **Nuclear Features:** The nuclei are relatively uniform in size and shape.
    * **Cytoplasm:** The cytoplasm appears eosinophilic (pink).
* **Arrangement of Epithelial Cells:** The epithelial cells are arranged in a single layer (simple epithelium) and are organized around the duct lumen.

Stroma:
* **Stromal Composition:** The stroma surrounding the epithelial structures appears to be composed of collagen fibers and fibroblasts.
* **Stromal Cellularity:** The stroma is sparsely cellular.
* **Inflammation:** There appears to be some inflammatory cells present in the stroma.

Other Features:
* **Vascularity:** A small blood vessel is visible, indicating vascularity of the tissue.
* **Overall Architecture:** The overall architecture appears relatively organized, with well-defined ducts surrounded by stroma.

Possible Interpretations:
Based on these attributes, the image could represent a normal or benign glandular tissue, or possibly a low-grade adenocarcinoma. Further evaluation would require examining more tissue samples, assessing for signs of cellular atypia (abnormal cell morphology), high mitotic rate (cell division), and invasion into the surrounding stroma.
Important Note: This is a limited assessment based on a single image. A definitive diagnosis requires a comprehensive evaluation by a qualified pathologist.

==

Description 3:
Here are some attributes that this pathology image shows:
* **Epithelial cells:** A layer of cells exhibiting a columnar or cuboidal shape, characterized by a basally located nucleus.
* **Lumen:** An open, empty space or cavity within a tubular structure, formed by the arrangement of epithelial cells.
* **Connective Tissue Stroma:** The supporting tissue surrounding the epithelial cells, composed of fibrous elements and cells.
* **Hematoxylin and Eosin (H&E) staining:** The image appears to be stained with H&E, a common staining technique in histology that highlights cell nuclei in blue/purple and cytoplasm and extracellular matrix in pink.
* **Duct-like Structure:** The presence of a tubular structure formed by epithelial cells surrounding a lumen.
* **Cellular Nuclei:** Darkly stained, round or oval structures within the cells, representing the cell's genetic material.
* **Fibrous Texture:** The stroma shows a fibrous texture, indicating the presence of collagen fibers.
* **Glandular architecture:** Evidence of gland-like structures, with cells arranged around a central space or lumen.
* **Cellular density:** The degree of cell packing within the tissue.
* **Cytoplasmic staining:** The cytoplasm of the cells appears pink.
* **Well-defined cell borders:** Distinct boundaries around individual cells.

Fig. S4. See caption on the page after the next.

Description 1:
Okay, let's analyze the histology slide to determine if it's normal breast tissue or invasive carcinoma.

Normal Breast Tissue Assessment:
* **Architecture:** The image shows a duct-like structure. The acini (if present) appear to be round to oval, with some variation in size, but not exceeding a 2:1 ratio. The arrangement seems organized. There are no irregular nests, cords, or sheets of cells. The glandular structures are not severely distorted or fused.
* **Cell Morphology:** The epithelial cells appear relatively uniform in size and shape (cuboidal to columnar). The cytoplasm is generally abundant. It's difficult to definitively identify myoepithelial cells in this image, but the presence of a second cell layer is possible.
* **Nuclei:** The nuclei are generally round to oval with smooth contours. The chromatin distribution appears even in most cells. Nucleoli are not prominent. The nuclear-to-cytoplasmic ratio is relatively low. Nuclei are polarized towards the basement membrane in most areas. There is no significant nuclear pleomorphism, hyperchromasia, or irregular chromatin distribution in the majority of cells.
* **Stroma:** The stroma appears somewhat dense, but not overwhelmingly fibrotic. There are no prominent inflammatory cells visible.
* **Basement Membrane:** The basement membrane appears intact and well-defined around the ducts in most areas.

Invasive Carcinoma Assessment
* **Architecture:** There is no obvious loss of normal lobular architecture. There are no irregular nests, cords, or sheets of cells. There are no fused or distorted glandular structures to the point of being unrecognizable.
* **Cell Morphology:** There is no significant pleomorphism. The cells are not arranged in single file. The cells are not tightly packed together. It's difficult to definitively assess myoepithelial cell presence/absence.
* **Nuclei:** There is no significant nuclear pleomorphism, hyperchromasia, irregular chromatin distribution, prominent nucleoli, or irregular nuclear membranes. No mitotic figures are obvious. The nuclei are not remarkably large or irregular.
* **Stroma:** The stroma is not obviously desmoplastic. There is no evident angiogenesis.
* **Tumor Boundary:** This doesn't apply, as there is no clear tumor mass.
* **Invasion:** There is no definitive evidence of lymphatic or vascular invasion.
* **Necrosis:** There are no areas of cell death (necrosis).

Comparison and Conclusion:
Based on the analysis, the features are more consistent with **normal breast tissue** than invasive carcinoma. Crucially, there is *no definitive evidence of invasion*. The architecture is relatively organized, the cell morphology is largely uniform, and the nuclear features are not overtly malignant. The stroma is not markedly desmoplastic.

Conclusion: The probability of this being normal breast tissue is high. There are no clear indications of invasive carcinoma.
Confidence Level: High
==

Description 2:
Okay, let's analyze the histology slide based on the provided criteria.

Architecture:
* There appears to be a recognizable ductal structure. The duct is elongated and slightly irregular in shape, but it maintains a lumen. Acini are not clearly definable, but the cells are arranged in a somewhat organized fashion, suggesting a glandular structure.
* **Negative Criteria:** There are *no* clear irregular nests, cords, or sheets of cells, and the glandular structure is not severely distorted or fused.

Cell Morphology:
* The epithelial cells within the duct are generally cuboidal to low columnar. There is a moderate amount of cytoplasm, which appears somewhat variable in density. A second cell layer is not clearly defined.
* **Negative Criteria:** There is *no* significant pleomorphism (variation in cell size and shape) exceeding a 2:1 ratio.

Nuclei:
* The nuclei are generally round to oval, with relatively smooth contours. The chromatin appears evenly distributed in *most* cells. Nucleoli are not readily apparent. The nuclear-to-cytoplasmic ratio appears relatively low. Nuclei are polarized towards the basement membrane in *most* areas.
* **Negative Criteria:** There is *no* clear nuclear pleomorphism, hyperchromasia, irregular chromatin distribution, prominent nucleoli, or irregular nuclear membranes in the majority of cells.

Stroma:
* The stroma appears somewhat dense and fibrotic, but not overwhelmingly so. There is abundant stroma surrounding the duct. Scattered inflammatory cells (lymphocytes) are present.
* **Negative Criteria:** There is *no* overwhelming desmoplasia (dense fibrosis).

Basement Membrane:
* The basement membrane appears intact and relatively well-defined around the duct in *most* areas.

Invasive Carcinoma Assessment:
Based on the provided image, there is NO definitive evidence of invasive carcinoma:

* **Negative for:**
    * Irregular nests, cords, or sheets of cells.
    * Significant pleomorphism.
    * Nuclear pleomorphism, hyperchromasia, irregular chromatin distribution, prominent nucleoli, or irregular nuclear membranes.
    * Overwhelming desmoplasia.
    * Single-file arrangement of cells.
    * Lymphatic or vascular invasion.
    * Necrosis.

Comparison and Conclusion:
The features observed are most consistent with normal breast tissue, specifically a ductal structure within a moderately dense stroma. While there are some minor irregularities in the ductal shape and stromal density, there is NO definitive evidence of invasive carcinoma. The absence of significant pleomorphism, irregular architectural patterns, desmoplasia, and lymphatic/vascular invasion strongly suggests a benign diagnosis.

Conclusion:
The probability of normal breast tissue is HIGH.
Confidence Level: High.

Fig. S4. See caption on the following page

Description 3:
Okay, let's analyze the histology slide to determine if it represents normal breast tissue or invasive carcinoma based on the provided criteria.

Normal Breast Tissue Assessment:
* **Architecture:** The image displays structures resembling ducts and acini. There is a recognizable architectural arrangement with a visible duct. The duct appears elongated with a central lumen. The acini display round to oval shapes, and there is minimal variation in size. No irregular nests, cords, or sheets of cells are observed. The glandular structures are not fused or severely distorted.
* **Cell Morphology:** The epithelial cells in the duct are generally uniform, with slight variations in size and shape. The cells appear cuboidal to low columnar. The cytoplasm is generally abundant, and cells are arranged in an organized manner. A second cell layer is likely present.
* **Nuclei:** The nuclei are generally round to oval, with smooth contours. Chromatin distribution appears even in most cells. Nucleoli are not prominent. The nuclear-to-cytoplasmic ratio appears relatively low. Nuclei are polarized towards the basement membrane in most areas. No nuclear pleomorphism, hyperchromasia, irregular chromatin distribution, prominent nucleoli, or irregular nuclear membranes are observed.
* **Stroma:** The stroma appears loose to moderately dense. Abundant stroma surrounds the duct. Few inflammatory cells are present. No overwhelming fibrosis (desmoplasia) is observed.
* **Basement Membrane:** The basement membrane is intact and well-defined around the duct in most areas.

Invasive Carcinoma Assessment:
* **Architecture:** There is no clear loss of normal lobular architecture. No irregular nests, cords, or sheets of cells are observed. No fused or distorted glandular structures are observed.
* **Cell Morphology:** No significant pleomorphism is observed. The cells are not arranged in single file. The cells are not tightly packed together. Myoepithelial cells are identifiable in the duct.
* **Nuclei:** No nuclear pleomorphism, hyperchromasia, irregular chromatin distribution, prominent nucleoli, or irregular nuclear membranes are observed. No mitotic figures are present.
* **Stroma:** The stroma is not desmoplastic. There is no evidence of angiogenesis.
* **Tumor Boundary:** Not applicable, as there is no evidence of a tumor.
* **Invasion:** There is no definitive evidence of lymphatic or vascular invasion.
* **Necrosis:** No areas of cell death (necrosis) are observed.

Comparison and Conclusion:
The findings strongly suggest normal breast tissue. There is no definitive evidence of invasion. The architecture is organized, the cells are generally uniform, the nuclei are bland, and the stroma is not desmoplastic. There is no evidence of lymphatic or vascular invasion.

Conclusion: The histology slide likely represents normal breast tissue.
Confidence Level: High.

Fig. S4. Comparison of classifications and descriptions before and after two-phase learning. (A) Using the same prompt and image, we repeated the classification 10 times to assess diagnostic consistency. The optimized prompt reduced classification variance to nearly half that of the initial prompt. (B) Examination of image descriptions (three examples shown) revealed that the optimized prompt produced more structured and detailed accounts, with greater coherence across descriptions, compared to those generated with the initial prompt.

Pathology Image description evaluation questionnaire:
Below are the LLM's descriptions of a pathology image. Do you think the LLM-generated descriptions accurately or correctly describe the corresponding images? Give some comments or feedbacks on how you think of the instruction and descriptions.

1. Here is an analysis of the breast tissue image:

2. Cellular organization: The cells are organized into distinct tubular structures. The tubules are lined by a single layer of epithelial cells. There is no …(rest not shown)

3. Pathology image

Pathology image

4. Rate the above description.*

1	2	3	4	5
☆	☆	☆	☆	☆

5. Do you have any comments on this description?

Instruction & Descriptions evaluation 1
You are given one instruction defining effective metrics for assessing pathology images to accurately diagnose normal vs. invasive carcinoma. There are several descriptions of pathology images generated by LLM's according to the given instruction. You may go through the instruction, images and their corresponding descriptions.
Do you think the instruction is effective in pathology dignosis or not? Do you think the LLM-generated descriptions accurately or correctly describe the corresponding images? Give some comments or feedbacks on how you think of the instruction and descriptions. If they are ineffective, how would you modify them to make them better?
You may add comments under each description. You can also leave an overall comment or suggestion at the end of the survey.

1. Instruction: Examine the provided breast tissue image, paying close attention to the following characteristics … …(rest not shown)

2. Pathology Image

Pathology image

3. Image 1 Description: Here's an analysis of the breast tissue image:
Cellular architecture: The image shows a well-defined structure, a duct-like formation lined by cells. The cells are organized in a single layer, forming a distinct epithelial lining. There are no obvious regions with aberrant cell grouping or unusual formations … (rest not shown)

Fig. S5. Templates of the pathologist evaluation and feedback questionnaires. Two distinct questionnaires were administered to gather assessments and qualitative feedback from board-certified pathologists. The top questionnaire presented a pathology image alongside an AI-generated description and asked respondents to rate the description on a five-point Likert scale. Sixteen descriptions were evaluated. The bottom questionnaire solicited open-ended comments on both the prompts and the corresponding AI-generated descriptions. These qualitative responses were subsequently utilized for Expert Knowledge Augmentation.

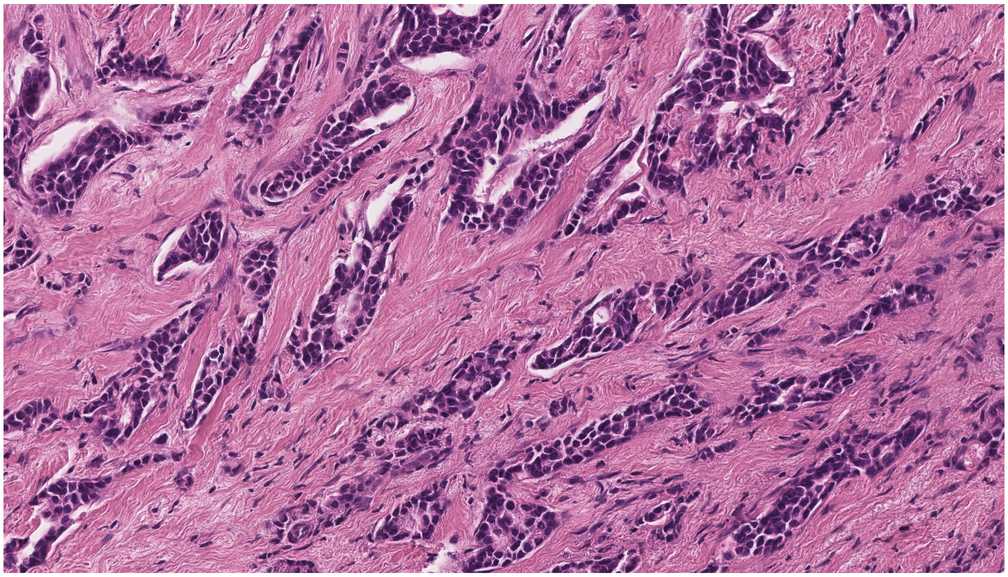

RECAP-PATH description:

- Cellular Architecture: The cells are not organized into well-defined structures like ducts or lobules. They appear randomly distributed and **lack a cohesive pattern**. There are regions with aberrant cell grouping and unusual formations, suggesting a lack of normal tissue organization.
- Cell Morphology: The cells exhibit significant variation in size and shape (pleomorphism). Nuclei are large, irregular, and exhibit hyperchromasia (darker staining), indicating abnormal nuclear features.
- Cell Proliferation: **Multiple mitotic figures are visible**, indicating increased cell division. Some mitotic figures appear abnormal in structure, with atypical spindle-shaped or multipolar configurations.
- Connective Tissue: The stroma appears dense and fibrous, suggesting an increase in connective tissue. **There is evidence of neovascularization** (new blood vessel formation), which is a common feature of invasive tumors.
- Categorization: Based on these observations, the image is most likely invasive carcinoma. The absence of normal tissue organization, abnormal cell morphology, increased cell proliferation with atypical mitosis, and dense stroma with neovascularization are all strong indicators of malignancy.

Pathologist 1 Feedback:

- Cellular Architecture: The description is acceptable but lack precision. Focal tubular/ductal structures are present. The tumor cells are arranged in irregularly shaped tubules or cords with an infiltrative growth pattern. The term "cohesive" is typically used to describe the tendency of cells to adhere to one another. In this view, although the ducts and cords are distributed irregularly, the cells themselves remain cohesive.
- Cell Morphology: The description is generally accurate. However, the concept of pleomorphism is somewhat subjective or relative. Nuclear pleomorphism in breast cancer is typically graded on a three-tier scale. In my assessment, this case corresponds to grade 2 (moderate) pleomorphism.
- Cell Proliferation: A few areas appear suggestive of mitosis. However, mitotic figures are typically assessed under higher magnification. At this resolution, I cannot confidently confirm the presence of atypical mitosis.
  Connective Tissue: No neovascularization is observed in this view.

Pathologist 2 Feedback:

- Cellular Architecture: The cells are randomly distributed (infiltration), not organized into well-defined structures. There are regions with aberrant cell grouping and unusual formations, suggesting a lack of normal tissue organization.
- There is no obvious blood vessel in this image. (not helpful)

Pathologist 3 Feedback:

- Good, except that I don't see any mitotic figure in the image. I do not see any neovascularization.

Fig. S6. Example of expert pathology feedback. In this example, specific portions of the image description flagged by pathologists are highlighted. Feedback from three pathologists was collected and incorporated into the learning framework.

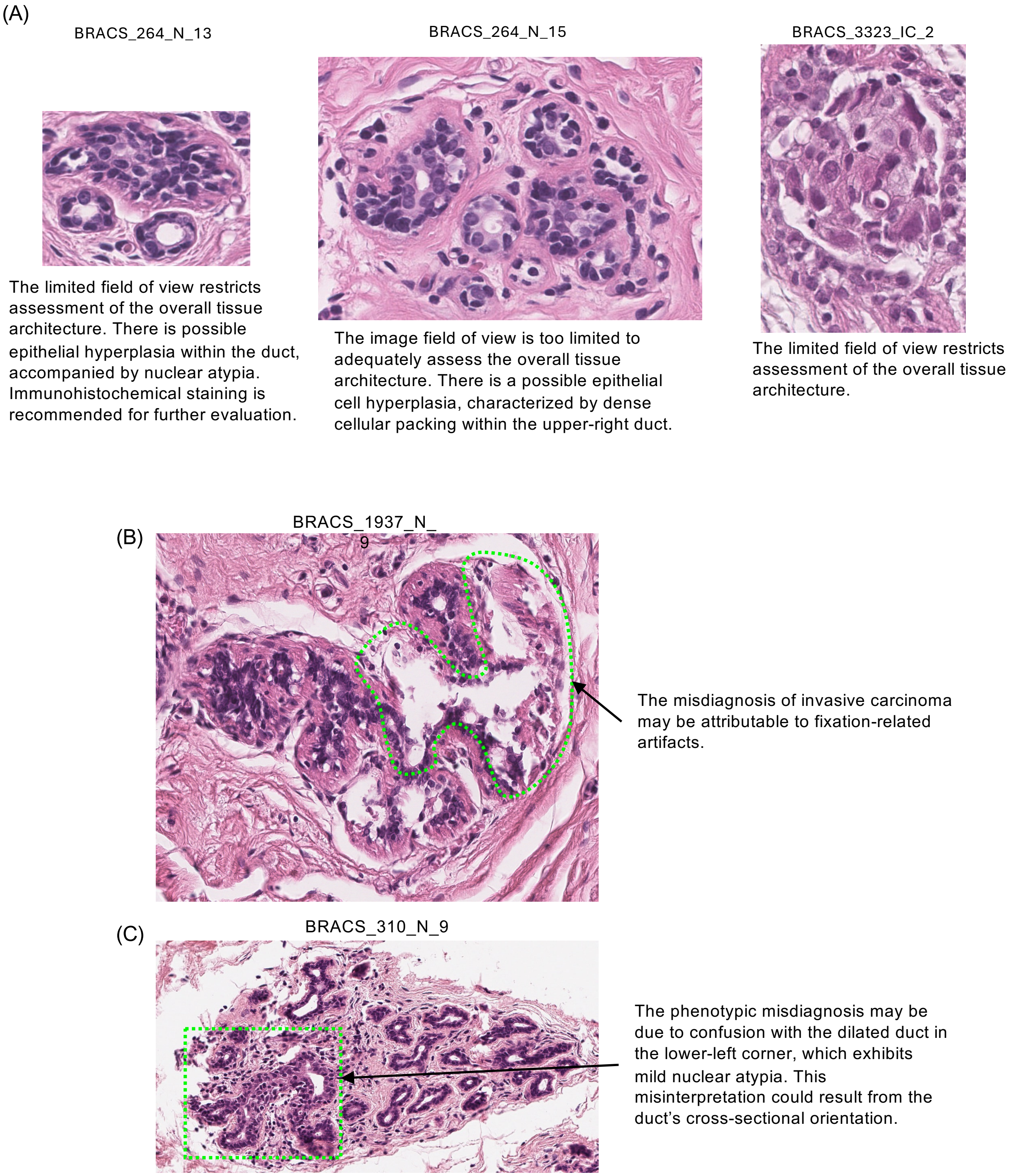

Fig. S7. Representative examples of misclassification. Review of misclassified images revealed three recurring patterns that contributed to diagnostic errors. (A) Limited field of view restricted evaluation of overall tissue architecture; within the duct, possible epithelial hyperplasia with nuclear atypia and dense cellular packing was noted. (B) Some misclassifications of invasive carcinoma were attributable to fixation-related artifacts. (C) Phenotypic misdiagnosis often arose from confusion with a dilated duct in the lower-left corner, which exhibited mild nuclear atypia; the duct's cross-sectional orientation further contributed to this misinterpretation.
