# Supplementary material for: Adaptive diagnostic reasoning framework for pathology with multimodal large language models
Source: Commun Med (Lond). 2026 Mar 7;6:236. doi: 10.1038/s43856-026-01491-z (PMC13096528; doi:10.1038/s43856-026-01491-z)
Supplement: Supplementary file 3 — Description of Additional Supplementary files [file 43856_2026_1491_MOESM3_ESM.docx]

**Description of Additional Supplementary Files**

File name: Supplementary Data

Description: Source data for Figures 2A–C, 3B, 5B, and 5D as well as Python code with embedded raw input data for reproducing Figures 2F, 4D, and 4F
